# Effects of microwave radiation on brain energy metabolism and related mechanisms

**DOI:** 10.1186/s40779-015-0033-6

**Published:** 2015-02-17

**Authors:** Yan-Hui Hao, Li Zhao, Rui-Yun Peng

**Affiliations:** Beijing Institute of Radiation Medicine, Beijing, 100850 China

**Keywords:** Microwave radiation, Brain, Energy metabolism, Mitochondria, Mechanisms

## Abstract

With the rapid development of electronic technologies, anxiety regarding the potential health hazards induced by microwave radiation (MW) has been growing in recent years. The brain is one of the most sensitive target organs for microwave radiation, where mitochondrial injury occurs earlier and more severely than in other organs. Energy metabolism disorders do play an important role during the process of microwave radiation-induced brain damage. In this paper, we will review the biological effects of microwave radiation, the features of brain energy supply and consumption and the effects of microwave radiation on mitochondrial energy metabolism and potential related mechanisms.

## Introduction

Microwaves (MW), electromagnetic waves with frequencies ranging from 300 MHz to 300 GHz, have been widely used in the telecommunications, agriculture, transportation, medical and military fields. The popularization of mobile phones, computers, household appliances and other electronic equipment has made learning, working and accessing entertainment much more convenient. With the intensive development of various advanced military weaponry equipment, such as early warning aircraft, electronic jammers and new radar, soldiers are always exposed to intricate environmental factors, including intensive and complex MW radiation. As the fourth largest source of pollution after air, water and noise, MW radiation induces many biological effects [1]. The brain is the most sensitive target organ for MW radiation, where mitochondrial injury occurs earlier and more severely than in other organs. Studies on the effects of MW radiation on brain energy metabolism have aroused great concern.

## Review

### Biological effects of MW radiation

The biological effects of MW radiation fall into two types: thermal and non-thermal effects [2,3]. Both are present, with thermal effects prominent in the case of high-power and high-frequency MW radiation and non-thermal effects predominant in the case of low-power MW radiation [4]. MW radiation has multi-faceted effects on many systems within an organism, including the nervous [5-7], endocrine [8], cardiovascular [9], immune [10,11], reproductive [12-14] and hematopoietic [15] systems. The brain always requires a high rate of oxygen and energy consumption to maintain regular functions. Therefore, this organ is sensitive to non-infectious stimuli such as ionizing radiation and hypoxia [16,17]. Research from our group and from others has demonstrated that microwave radiation damages hippocampal structures in rats, impairs long-term potentiation, decreases neurotransmitter concentrations, reduces synaptic vesicles in number and results in memory impairment [5,18,19]. Thus, the brain is generally accepted as the most sensitive target organ for MW radiation.

The damaging effects of MW radiation on the brain include brain dysfunction and brain structural damage. An epidemiological survey found that MW radiation caused human fatigue, headache, excitement, dreams, memory loss and other symptoms of neurasthenia [20]. In addition, there were impaired learning and memory abilities in rats after MW radiation, as determined by the Morris water maze [5,6,21,22]. MW radiation may also lead to neuronal shrinkage, nuclear condensation, mitochondrial swelling, an expanded endoplasmic reticulum, alterations to the synaptic gaps and widened vascular endothelial connections, where mitochondrial injury occurred earlier and more severely [5,21,23-25].

### Features of brain energy metabolism

In the human body, the brain has the greatest demand for oxygen and is susceptible to disturbances in energy metabolism, which is determined by its high metabolic rate, high oxygen consumption and low energy reserves. Mitochondria are the key sites of oxidative phosphorylation (OXPHOS) and the synthesis of adenosine triphosphate (ATP). The redox enzymes and the coenzymes involved in the respiratory chain lie in the mitochondrial inner membrane in close proximity. Electrons passing through the respiratory chain drive protons from the matrix side to the cytoplasmic side across the mitochondrial inner membrane. When protons reflux along the concentration gradient, the energy released is used by ATP synthase to catalyze ATP synthesis.

In addition to energy conversion, mitochondria also play other important roles, such as in the regulation of apoptosis and Ca^2+^ storage. Mitochondria are not only the starting point of many signal transduction pathways but also the target.

### Effects of MW radiation on mitochondrial energy metabolism

MW radiation is detrimental to brain energy metabolism. Intrinsically, neurons are extremely sensitive to a reduced ATP availability. As the main source of energy, mitochondria are prone to MW radiation-induced injury. Wang *et al*. [26] exposed monkeys to MW radiation with average power densities of 5 mW/cm^2^ and 11 mW/cm^2^ for 10 s and 4.68 μW/cm^2^ for 12 h/d for 30 d cumulatively. Abnormalities in mitochondrial function-related metabolites in urine, such as succinic acid, citric acid and 2-keto-glutaric acid, were induced after a single radiation event of 5 mW/cm^2^ and 11 mW/cm^2^ and after a long-term radiation of 4.68 μW/cm^2^, revealing by metabolomics the hypersensitivity of mitochondria to MW radiation.

### Effects of MW radiation on mitochondrial structure

MW radiation leads to mitochondrial structural damage, primarily observed as mitochondrial swelling and cavitation and disorganized, broken and sparse cristae.

To some extent, MW radiation affects mitochondria structurally in a dose-dependent manner. Zhao *et al*. [5] exposed male Wistar rats to MW radiation with average power densities of 2.5, 5 and 10 mW/cm^2^, with the specific absorption rates (SAR) of 1.05, 2.1 and 4.2 W/kg, respectively, for 6 min/d for 30 d. In the hippocampus of the MW-exposed rats, the mitochondria were swollen and vacuolized, and the cristae were disordered and fewer in number. In addition, these ultrastructural changes in the mitochondria tended to be more severe relative to the increasing SAR. Xie *et al*. [27] exposed male Wistar rats to MW radiation for 1 h at average power densities of 3 and 30 mW/cm^2^, respectively. No significant changes occurred in the mitochondria of the hippocampus or cerebral cortex in the 3 mW/cm^2^ group, while the mitochondria in the 30 mW/cm^2^ group did become damaged. These results suggest that, within a certain range, the degree of mitochondrial structural damage positively correlates with the dose of MW radiation.

MW radiation damaging mitochondrial structures obeys a time-response relationship. Xie *et al*. [27] exposed male Wistar rats to MW radiation (30 mW/cm^2^, duration: 1 h). Immediately after radiation, the mitochondrial ultrastructure showed a slight disturbance in the rat hippocampus and cerebral cortex; 3 h after radiation, the visible swelling of the mitochondria increased significantly and cristae became disorganized, broken and sparse; 24 h after radiation, mitochondrial degeneration was observed, demonstrated by myelin-like structures and occasional dense deposits in the mitochondria. In short, ultrastructural changes in the rat brain mitochondria were induced within 24 h of the post-30 mW/cm^2^ MW radiation exposure.

Long-term and low-dose cumulative MW radiation leads to significant damage in mitochondria. Dong *et al*. [21] exposed SD rats to MW radiation (4.68 μW/cm^2^, 12 h/d, duration: 30 d), which resulted in similar structural changes, such as swelling and cavitation in the mitochondria of the radiation-exposed rat hippocampus and cerebral cortex.

### Effects of MW radiation on mitochondrial energy metabolism

#### Reduced ATP content

As the “cell power plant”, the most important function of mitochondria is to provide energy for the cell; therefore, intracellular ATP content is one of the most direct and objective indicators in the evaluation of mitochondrial function. In addition, ATPases hydrolyze ATP to ADP and release the energy stored in ATP.

Certain doses of MW radiation cause reduction in mitochondrial ATP synthesis. Zhao *et al*. [25] exposed male Wistar rats to pulsed MW radiation (30 mW/cm^2^, duration: 5 min). The results showed that the content of mitochondrial ATP in the hippocampus of MW-exposed rats dropped to the lowest levels 3 d after radiation and recovered 7 d after radiation, while the activity of the ATPases was greatly enhanced 3 d after radiation and recovered 7 d after radiation, suggesting a compensatory role played by this negative feedback regulation. Sander *et al*. [28] exposed SD rats to MW radiation with a frequency of 591 MHz at an average power density of 13.8 mW/cm^2^, which induced a reduced availability of ATP, resulting in brain energy metabolism disorders.

#### Decreased succinate dehydrogenase (SDH) activity

As one of the key enzymes of mitochondrial energy metabolism, SDH binds to the mitochondrial inner membrane and catalyzes the dehydrogenation of succinate to generate ATP ultimately, forming a bridge between the Krebs cycle and OXPHOS.

MW radiation reduces the activity of SDH. Zhao *et al*. [25] exposed male Wistar rats to pulsed MW radiation (30 mW/cm^2^, duration: 5 min). The SDH activity of the MW-exposed rat hippocampus decreased significantly 6 h after radiation, resulting in abnormalities in mitochondrial energy metabolism. Wang *et al*. [29] exposed Wistar rats to high power microwave (HPM) radiation of 10, 30 and 100 mW/cm^2^ for 5 min, respectively. They also found reduced SDH activity present in every exposure group, which recovered 7 d after radiation. Another study exposed male Wistar rats to MW radiation of 30 mW/cm^2^ for 15 min. The SDH activity of the MW-exposed rat hippocampus did not change significantly at 14 d after radiation, indicating that the MW radiation-induced decline in SDH activity is reversible under certain conditions [23].

#### Suppressed cytochrome c oxidase (COX) activity

COX is embedded in the mitochondrial inner membrane and is the terminal complex of the mitochondrial electron transport chain. As another one of the key enzymes of mitochondrial energy metabolism, COX is the only enzyme to transport electrons to oxygen to produce H_2_O and ATP [30,31]. It is believed that 90% of intracellular molecular oxygen is utilized by COX [32].

Certain doses of MW radiation negatively impact the activity of COX. Wang *et al*. [33] exposed primary cultures of cerebral cortical neurons of Wistar rats to continuous MW radiation of 900 MHz, with SARs of 0.38, 0.76, 1.15, 2.23 and 3.22 W/kg, respectively, for 2 h/d for 4 to 6 d. The results showed that the toxic effects of MW radiation on COX activity accumulated and that there was a dose-dependent relationship. Xiong *et al*. [34] used MW radiation of 30 mW/cm^2^ to irradiate male Wistar rats. The decreased COX activity and the reduced expression of COX I/IV mRNA and COX I protein were found after MW radiation, illustrating that MW radiation impacted COX activity at multiple levels.

### Potential mechanisms involved in MW radiation-induced disturbances in mitochondrial energy metabolism

By the rapid development of modern molecular biology techniques, studies on the mechanisms of the biological effects of MW radiation have been possible at the cellular and molecular levels. This section will review the potential mechanisms of MW radiation-induced brain energy metabolism disorders from seven aspects, including gene expression, the mitochondrial membrane, apoptosis, oxidative stress (OS), Ca^2+^ overload, mitochondrial DNA and the involved signal transduction pathways.

### Altered gene expression in the respiratory chain

MW radiation causes abnormal expression of the genes encoding the respiratory chain, resulting in brain energy metabolism disorders. Zhao *et al*. [35] exposed male Wistar rats to pulsed MW radiation (30 mW/cm^2^, duration: 5 min). There were multiple genes differentially expressed 6 h after radiation in the rat hippocampus (upregulated: syn1, ptprj, CD74 and MHCII; downregulated: ttr, enpp2, folr1, cdh22, spata2, spp1, calb2, tacl and dnpi), some of which (syn1, ttr and enpp2) are closely related to the metabolic function of mitochondria. As the neural metabolic marker, COX contains 13 subunits, with COX I-III encoded by mitochondrial genes and the other 10 subunits encoded by nuclear genes. COX I constitutes the catalytic center, and COX IV regulates the enzyme activity responding to ATP/ADP content [36,37]. Zhao *et al*. [38] found that exposing male Wistar rats to pulsed MW radiation (30 mW/cm^2^, duration: 5 min) reduced the expression of COX I/II mRNA 6 h after radiation and increased the expression of COX IV mRNA in 1 d, both of which tended to recover in 3 to 7 d, demonstrating that reduced COX activity in the rat hippocampus occurred after MW radiation of 30 mW/cm^2^. Xie *et al*. [39] exposed rats to acute MW radiation for 1 h at average power densities of 3 mW/cm^2^ or 30 mW/cm^2^, respectively. After MW radiation of 3 mW/cm^2^ for 0, 3 and 24 h, no significant changes in the COX I and COX IV mRNA expression levels in the rat cerebral cortex and hippocampus were found. However, after MW radiation of 30 mW/cm^2^ for 0, 3 and 24 h, COX I mRNA expression in the rat cerebral cortex and hippocampus decreased significantly, but no significant change in COX IV mRNA expression levels was found. In conclusion, MW radiation downregulates the COX I gene encoded by mitochondrial DNA in the cerebral cortex and hippocampus of rats in a dose-dependent manner. These results suggest that the changes in gene expression caused by MW radiation are important factors in mitochondrial dysfunction and brain energy failure.

### Damaged mitochondrial membrane

The mitochondrial membrane enables the mitochondria to be relatively independent and to maintain homeostasis its internal environment and plays important roles in energy conversion, signal transduction and material transport. A variety of enzymes closely related to energy metabolism, such as SDH and complex I-IV, bind to the mitochondrial membrane. As a key part of the synthesis of ATP, a damaged mitochondrial membrane leads to a decreased activity of complex I/III and to further disturbances in energy metabolism [40].

The structural damage of the mitochondrial membrane is one of the most important mechanisms of MW radiation-induced disturbance of brain energy metabolism. Mitochondria are organelles wrapped by a double membrane, with the inner membrane forming cristae, which increase the surface area of the mitochondrial membrane greatly. As biofilms are targets of electromagnetic radiation [41], it can be inferred that the structural characteristics of mitochondria determine its high sensitivity to MW radiation-induced injury.

There are multiple possible ways through which MW radiation may structurally damage the mitochondrial membrane. First, MW radiation has the ability to enhance molecular rotation and vibration and to increase the collision frequency between molecules, leading to the breaking of chemical bonds and thus, damage to the mitochondrial membrane structure [42]. Second, MW radiation leads to a significant increase in intracellular reactive oxidative species (ROS) and the disorder of antioxidant enzymes, causing oxidative modification of biological macromolecules and mitochondrial damage [22,43-46]. Third, MW radiation causes intracellular Ca^2+^ overload and induces mitochondrial membrane injury through the activation of phospholipases and proteases [47-49].

### Apoptotic death of neural cells

During the process of MW radiation-induced brain damage, apoptosis is one of the final outcomes of damaged cells. Blocking apoptosis to relieve the effect of MW radiation on the nervous system and to find new targets for prevention and treatment is of great value.

MW radiation induces neural cell apoptosis *via* the classical mitochondria-dependent caspase-3 pathway. Zuo *et al*. [50] exposed PC12-derived neuron-like cells and Wistar rats to 2.856 GHz for 5 min and 15 min, respectively, at an average power density of 30 mW/cm^2^. The results showed chromatin condensation and apoptotic body formation in neural cells 6 h after MW exposure. Moreover, the mitochondrial membrane potential (MMP) decreased, and DNA fragmentation increased, leading to an increase in the percentage of apoptotic cells. Furthermore, the ratio of Bax/Bcl-2 and the expression of cytochrome c, cleaved caspase-3 and PARP all increased. Kesari *et al*. [43] exposed 45-day-old male Wistar rats for 2 h a day for 60 d by mobile phone to investigate the effect of 3G cell phone exposure. The results showed that MW radiation emitted from the 3G mobile phone significantly induced DNA strand breaks in the brain. Meanwhile, significant increases in micronuclei, caspase-3 and apoptosis were also observed in the exposed group. Mitochondrial dysfunction-mediated cytochrome c release and the subsequent activation of caspases were found, which were involved in the process of radiation-induced apoptotic cell death.

### Oxidative stress

MW radiation activates the NADH oxidase-mediated increase in ROS, and in turn, excessive ROS damages the mitochondrial electron transport chain, which is the main source of ROS, ultimately forming a vicious cycle and aggravating the disturbance in brain energy metabolism [13,51-54]. Deshmukh *et al*. [22] subjected Fischer-344 rats to MW exposure (frequency of 900 MHz; SAR of 8.4738 × 10^−5^ W/kg) in a gigahertz transverse electromagnetic cell (GTEM) for 30 days (2 h/d, 5 d/week). The results showed a significant increase in OS, as evidenced by the increase in levels of MDA (a marker of lipid peroxidation), protein carbonyl (a marker of protein oxidation) and unaltered glutathione (GSH) content in the blood. Thus, the study demonstrated that low-level MW radiation was capable of leading to OS. Kesari *et al*. [44] exposed 35-day old Wistar rats to a mobile phone for 2 h per day for a duration of 45 d, where the SAR was 0.9 W/kg. The results indicated a significant increase in the level of ROS, a significant decrease in the levels of glutathione peroxidase (GPx) and superoxide dismutase (SOD), and an increase in catalase (CAT) activity. In addition, it is reported that inhibiting OS and removing ROS had a large therapeutic effect on MW radiation-induced brain damage [55,56]. Taken together, excessive ROS plays an important role during the process of MW radiation-induced injury to brain energy metabolism.

Excessive ROS is detrimental to brain energy metabolism. First, excessive levels of ROS-induced DNA breakage (nuclear and mitochondrial DNA) may be one of the key reasons for MW radiation-induced brain energy metabolism disorders [43,44,57-59]. Kesari *et al*. [43] had 45-day-old male Wistar rats exposed for 2 h a day for 60 d to a mobile phone and found that the ROS content showed a positive linear correlation with DNA damage. Another study showed that pretreatment with radical scavengers was capable of blocking MW radiation-induced DNA damage [59]. Second, excessive levels of ROS were closely related to neural cell apoptosis, as previously described [43,44]. Third, as a second messenger, increased ROS-induced excessive activation of one or more signaling pathways is believed to play a more important role in cell damage rather than in oxidative modification [60,61]. The role of ROS in MW radiation-induced brain damage needs to be further explored.

### Ca^2+^ overload

Under normal circumstances, the extracellular free Ca^2+^ concentration is much higher than the intracellular concentration, and more than 90% of intracellular Ca^2+^ is stored in the endoplasmic reticulum and mitochondria. Therefore, a slight influx of Ca^2+^ is able to create a sharp rise in the concentration of cytoplasmic Ca^2+^ and trigger a series of physiological responses.

Increased cytoplasmic Ca^2+^ exists during the process of MW radiation-induced brain damage. Yang *et al*. [47] exposed primary cultures of hippocampal neurons of rats to MW radiation for 5 min at an average power density of 10 mW/cm^2^. The results showed a significant increase in cytoplasmic Ca^2+^ immediately after radiation. Lu *et al*. [48] exposed primary cultures of glial cells to 2450 MHz for 2 h/d for 3 d at an average power density of 4 mW/cm^2^. An increased intracellular free Ca^2+^ was also found.

Ca^2+^ overload leads to brain energy metabolism disorders. Excessive activation of the mitochondrial permeability transition pore (mPTP), caused by Ca^2+^ overload, may be one important reason. Studies have reported that Ca^2+^ overload-induced activation of mPTP results in mitochondrial swelling and fragmentation [62,63]. In addition, when mPTP is activated excessively, the mitochondrial membrane permeability increases, MMP disappears, the respiratory chain is uncoupled from OXPHOS and ATP synthesis ceases [64,65]. Additionally, Ca^2+^ is an important intracellular second messenger that is able to activate a variety of signaling molecules such as PKC, AC and cAMP-PDE. The role played by Ca^2+^ during MW radiation-induced mitochondrial injury deserves more in-depth research.

### Impaired mitochondrial DNA

Mitochondrial DNA (mtDNA) encodes 13 subunits of the respiratory chain complex and 22 tRNA and 2 rRNA of mitochondria, and is of the utmost importance to OXPHOS and ATP synthesis. Mitochondrial transcription factor A (mtTFA), a key factor encoded by nuclear genes involved in the regulation of mtDNA, plays important roles in the integrity, self-replication and repair of mtDNA after being transported from the cytoplasm to the mitochondria [66].

MW radiation can break mtDNA or change the expression of mtDNA, resulting in decreased ATP production. First, mtDNA, with the structure of a double helix ring, lacks the protection of protein binding and repair systems and is much more susceptible to external stimuli, such as MW radiation, than nuclear DNA is. MW radiation is capable of breaking nuclear DNA strands [43,44,57-59]. In addition, ROS has the ability to induce mtDNA mutations and create barriers in OXPHOS and ATP generation [67]. However, the effects of MW radiation-induced mtDNA damage on brain energy metabolism still require further study. Second, mtTFA needs to be properly transferred from the cytoplasm to the mitochondria to function, which leads to mitochondrial dysfunction when this process is disturbed by MW radiation. Xie *et al*. [27] exposed male Wistar rats to MW radiation (30 mW/cm^2^, duration: 1 h). The expression of mtTFA mRNA in the rat hippocampus and cerebral cortex increased, responding to the reduced ATP content within a possible negative feedback regulation. Xu *et al*. [68] exposed primary cultures of cortical neurons of neonatal rats to MW radiation (frequency and power density unknown). The expression of mtTFA mRNA and protein increased, but new mtTFA did not inhibit the impact of MW radiation on energy metabolism. In another study, these authors confirmed the inhibitory effect of MW radiation on the transport of mtTFA from the cytoplasm into the mitochondria by using isotope-labeling technique, which may be the primary reason for the MW radiation-induced ATP decrease [69].

### Signaling pathways involved

There are many signaling pathways involved in the process of MW radiation-induced mitochondrial dysfunction, including the phosphatidylinositol 3-kinase (PI3K) pathway and the mitogen-associated protein kinase (MAPK) pathway, which are adaptive responses of cells that regulate cellular functions and promote their survival.

#### PI3K/Akt pathway

There is enough evidence that the PI3K/Akt pathway, an anti-apoptotic prosurvival kinase signaling cascade, plays a pivotal role in cellular survival [70,71]. Hypoxia inducible factor-1α (HIF-1α), a key physiological sensor of oxygen level in most mammalian cells, plays an important role in cellular survival, glucose metabolism and transport and metabolic adaptation by regulating the expression of its target genes [72-75]. In addition, it has been shown that activation of HIF-1α by the PI3K/Akt/mTOR signaling pathway plays an important role in neuroprotection [76,77].

Great importance has been attached to the PI3K/Akt signaling pathway during the process of MW radiation-induced brain damage, and the activation of HIF-1α, a key target molecule of PI3K/Akt pathway, is capable of restoring the impaired mitochondrial energy metabolism caused by MW radiation to a certain extent. Wang *et al*. [78] exposed rats to acute MW radiation for 5 min at average power densities of 30 and 100 mW/cm^2^, respectively. The expression of HIF-1α mRNA and protein in the rat hippocampus and cerebral cortex increased significantly at 6 h to 1 d after radiation. Another study had male Wistar rats exposed to pulsed MW radiation for 30 d (6 min/d, 5 d/week) at average power densities of 2.5, 5 and 10 mW/cm^2^, respectively. The expression of HIF-1α mRNA and protein in the rat hippocampal neurons increased at 14 d to 1 month after radiation in the 2.5 and 5 mW/cm^2^ groups, but decreased in the 10 mW/cm^2^ group [79]. Thus, HIF-1α upregulation occurs after both single acute and long-term MW radiation. However, it is not clear whether the activated HIF-1α is helpful to the cells impaired by MW radiation. Zhao *et al*. [24] exposed PC12-derived neural-like cells to MW radiation of 30 mW/cm^2^ for 5 min. HIF-1α overexpression protected mitochondria from injury by increasing ATP and MMP levels, while HIF-1α silencing promoted MW-induced mitochondrial damage. PI3K signaling activation was required for the MW-induced HIF-1α activation and protective response. Although HIF-1α represents a promising therapeutic target for MW radiation injury, how HIF-1α is regulated and what its effective targets in the mitochondria may be remain unknown.

#### MAPK pathway

MAPK, which is composed of different gene products terminating in a variety of transcription factors involved in survival, proliferation and cell death, depending on the strength of the stimulus, regulates the balance between cell survival/differentiation and cell death/apoptosis [80]. The three subfamilies of MAPK include extracellular regulated protein kinase (ERK), c-jun N-terminal kinase/stress-activated protein kinase (JNK/SAPK) and p38MAPK.

ERK signaling-mediated upregulation of HIF-1α protects from mitochondrial dysfunction caused by MW radiation. Zhao *et al*. [24] exposed PC12-derived neural-like cells to MW radiation of 30 mW/cm^2^ for 5 min. Inhibition of p-Erk1/2 promoted a MW-induced decrease of ATP and MMP levels and induced a decreased expression of HIF-1α, demonstrating that ERK signaling was involved in the protective mechanism against MW-induced mitochondrial injury.

p38MAPK, known as cell death MAPK signaling, is involved in MW radiation-induced neural cell apoptosis. p38MAPK induces apoptosis by regulating the conformational changes and subsequent oligomerization of Bax, the dissipation of MMP and the cytochrome c release from mitochondria [81]. Kesari *et al*. [43] exposed 45-day-old male Wistar rats for 2 h a day for 60 d to a mobile phone to investigate the effect of 3G cell phone exposure. They found that the 3G mobile radiation induced apoptosis in the brain by activation of p38MAPK, the pathway of principal stress response.

## Conclusion

To date, the damaging effects of MW radiation on mitochondrial structure and function have been recognized, and studies at the cellular and molecular level on the related mechanisms have also made advances, enabling a number of potential molecular targets for the prevention and treatment of MW radiation to be proposed.

The following issues are present in this sphere of research: (a) MW radiation-induced disturbance of brain energy metabolism involves numerous parameters, such as the dose, time and frequency, which need to be explored further; (b) the biological effects of MW radiation are widespread, involving varieties of signaling pathways, and the present review is confined to investigating single signaling pathways and unable to analyze the effects of cross-talk between the various signaling pathways; (c) there are no specific markers for evaluating MW radiation damage effects and no effective molecular targets for the prevention and treatment of their injuries; (d) the after effects of MW radiation-induced mitochondrial damage are still unclear, and its correlation with mitochondria-related neurodegenerative diseases, such as Alzheimer’s disease, requires further study. The lack of identical standards among different laboratories creates a barrier for further development and exchange of information.

Taken together, this review on the effects of MW radiation on brain energy metabolism and the associated regulation mechanisms, molecular markers, drug targets and prevention measures shows the need for continued research efforts in this area.

## Abbreviations

ATP: Adenosine triphosphate
CAT: Catalase
CoQ: Coenzyme Q
COX: Cytochrome c oxidase
ERK: Extracellular regulated protein kinase
FADH2: Reduced flavin adenine dinucleotide
GPx: Glutathione peroxidase
GSH: Glutathione
GTEM: Gigahertz transverse electromagnetic cell
HIF-1α: Hypoxia inducible factor-1α
HPM: High power microwave
JNK: c-jun N-terminal kinase
MAPK: Mitogen-associated protein kinase
MMP: Mitochondria membrane potential
mPTP: Mitochondrial permeability transition pore
mtDNA: Mitochondrial DNA
mtTFA: Mitochondrial transcription factor A
MW: Microwave
NADH: Reduced nicotinamide adenine dinucleotide
OS: Oxidative stress
OXPHOS: Oxidative phosphorylation
PI3K: Phosphatidylinositol 3-kinase
ROS: Reactive oxidative species
SAPK: Stress-activated protein kinase
SAR: Specific absorption rate
SDH: Succinate dehydrogenase
SOD: Superoxide dismutase
